# Modularized Extracellular Vesicles: The Dawn of Prospective Personalized and Precision Medicine

**DOI:** 10.1002/advs.201700449

**Published:** 2018-01-02

**Authors:** Shi‐Cong Tao, Shang‐Chun Guo, Chang‐Qing Zhang

**Affiliations:** ^1^ Department of Orthopaedic Surgery Shanghai Jiao Tong University Affiliated Sixth People's Hospital 600 Yishan Road Shanghai 200233 China; ^2^ Institute of Microsurgery on Extremities Shanghai Jiao Tong University Affiliated Sixth People's Hospital 600 Yishan Road Shanghai 200233 China

**Keywords:** drug delivery, extracellular vesicles, modular design, nanovesicles, nucleic acids, targeting

## Abstract

Extracellular vesicles (EVs) are ubiquitous nanosized membrane vesicles consisting of a lipid bilayer enclosing proteins and nucleic acids, which are active in intercellular communications. EVs are increasingly seen as a vital component of many biological functions that were once considered to require the direct participation of stem cells. Consequently, transplantation of EVs is gradually becoming considered an alternative to stem cell transplantation due to their significant advantages, including their relatively low probability of neoplastic transformation and abnormal differentiation. However, as research has progressed, it is realized that EVs derived from native‐source cells may have various shortcomings, which can be corrected by modification and optimization. To date, attempts are made to modify or improve almost all the components of EVs, including the lipid bilayer, proteins, and nucleic acids, launching a new era of modularized EV therapy through the “modular design” of EV components. One high‐yield technique, generating EV mimetic nanovesicles, will help to make industrial production of modularized EVs a reality. These modularized EVs have highly customized “modular design” components related to biological function and targeted delivery and are proposed as a promising approach to achieve personalized and precision medicine.

## 1 Introduction

**Figure 1:**
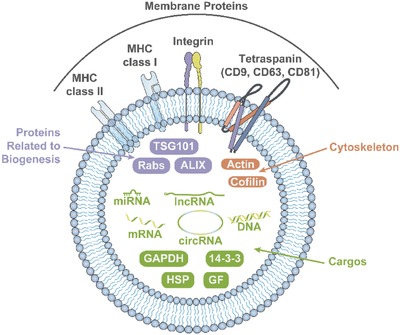
Structure and components of EVs.

In a variety of organisms, both prokaryotic and eukaryotic, almost all cells communicate with neighboring or distant cells through the secretion of extracellular vesicles (EVs), which consist of a lipid bilayer and membrane proteins that enclose proteins and nucleic acids, including messenger RNA (mRNA), microRNA (miRNA), and other noncoding RNAs (**Figure** **1**).^1^, ^2^, ^3^ According to their subcellular origin, EVs can be mainly classified into two categories: microvesicles (MVs, also known as ectosomes or microparticles, 100–1000 nm in diameter), which are released after formation by budding from the cytomembrane, and exosomes (Exos, 30–100 nm in diameter), which are produced inside multivesicular bodies (MVBs) and released after fusion of the MVBs with the cytomembrane.^4^, ^5^, ^6^ In addition, apoptotic bodies (800–5000 nm in diameter), which are shed into the extracellular environment from apoptotic cells, have several characteristics in common with MVs but are rarely involved in intracellular communication compared to MVs.^4^ One current hypothesis suggests that apoptotic bodies are promptly phagocytosed by phagocytes.^4^, ^7^

EVs were initially regarded as membrane debris without any real biological significance, but in 1996, Raposo et al.^8^ found that EVs participate in the immune response. Since then, EVs have attracted more and more attention as a natural method of intercellular communication.^5^

**Figure 2:**
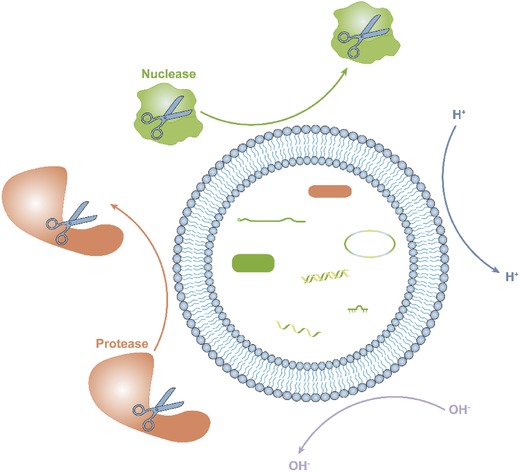
The isolating ability of the phospholipid layer protects the contents of EVs from exposure to components of the external environment, including acidic and alkaline conditions, proteases, and nucleases.

The tiny diameter of EVs provides several clinical benefits, including preventing vessel blockages,^9^ reducing phagocytosis by macrophages,^10^ and making injection easier. The structure of EVs also provides a number of therapeutic advantages in transferring information between cells. For example, EVs offer high physicochemical and biological stability,^11^ which may be due to the isolating ability of the phospholipid layer, protecting the contents of EVs from exposure to the external environment (**Figure** **2**).^12^ As a result, EVs can exist in almost all body fluids, including blood plasma,^13^ saliva,^14^ urine,^15^ bile,^16^ synovial fluid,^17^ semen,^18^ and breast milk.^19^ By expressing different surface proteins, EVs can have different targeting abilities.^20^ Because of these properties, EVs have become a popular focus in precision and personalized medicine.

It has already been demonstrated that EVs can be derived from almost all mammalian cell types, including healthy cells^21^, ^22^ and cancerous cells.^23^, ^24^ The composition of cancer‐derived EVs (C‐EVs) is markedly changed compared to EVs from healthy cells, and C‐EVs can therefore be used as diagnostic biomarkers of cancers.^25^, ^26^ As key players in the field of liquid biopsies, C‐EVs are potential biological markers that can detect the existence of tumor cells, even in very early stages, and can be used to monitor the progression of malignant tumors.^27^, ^28^ In addition to studies showing that EVs are characterized by specific membrane proteins,^29^ the nucleic acid contents of C‐EVs in the plasma/serum from malignant tumor patients are also markedly different than those from healthy controls, indicating that the specific nucleic acid content in C‐EVs should be the focus of greater attention in the search for new tumor biomarkers.^30^, ^31^ Recently, EVs have come to be considered effective diagnostic and predictive tools for patients suffering from malignant tumors, such as breast and prostate cancers.^32^, ^33^ In addition to tumors, EVs show potential in the diagnosis of infectious diseases and even in the assessment of disease progression.^34^, ^35^, ^36^

**Figure 3:**
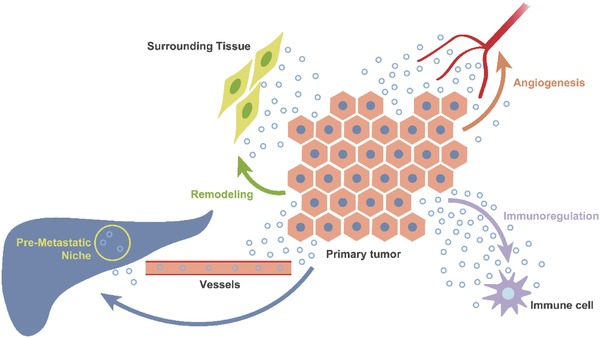
A suitable microenvironment created by C‐EVs. C‐EVs promote tumor progression by promoting angiogenesis, regulating immunity, and remodeling surrounding tissues, or laying the foundation for distant metastasis by forming a premetastatic niche.

In cancer progression (**Figure** **3**), C‐EVs can create a suitable microenvironment by promoting angiogenesis, regulating immunity, and remodeling surrounding tissue, or by laying the foundation for metastasis by forming a premetastatic niche.^37^, ^38^, ^39^, ^40^ It appears that cancers co‐opt EVs, a natural nanodelivery system, to transfer damaging information by altering the composition of EVs; this ability has inspired scientists to design novel drug therapeutics through reverse engineering.

The development of EV‐based medicine is a direct logical evolution from cell‐based therapies, especially stem cell therapies.^41^ Specifically, the therapeutic effects, which were once attributed to the direct participation of stem cells via proliferation and differentiation, are now known to be induced by a paracrine effect mediated by EVs.^41^ However, completely natural EVs may not be sufficient to provide treatments for all kinds of disease conditions; modified EVs have therefore been developed, indicating the beginning of the era of modularized EVs. A variety of modified EVs, including EVs specifically enriched in mRNA,^42^ miRNA,^12^, ^43^ and small interfering RNA (siRNA),^20^, ^44^ EVs with membrane modification,^45^, ^46^ and EVs carrying small molecule drugs^47^ and superparamagnetic iron oxide nanoparticles^48^ have been developed. As the era of personalized and precision medicine continues to develop, the demands of both the targeting ability toward pathological organization combined with limited damage to normal tissues^49^ to minimize side effects (precision medicine) and freely assembled agents based on a patient's personalized database^50^, ^51^ to maximize therapeutic effects (personalized medicine) will continually increase.

Recently, EV mimetic nanovesicles (EVMs), which have as much as a 120‐fold higher production yield compared to EVs from the same number of source cells, have been created by breaking down cells through serial extrusion.^52^ Furthermore, it has gradually been revealed that EVMs show similar properties to EVs;^53^, ^54^ therefore, the method of producing EVMs might be a much more effective way to manufacture modularized EVs.

In this review, we highlight and discuss the growing trend of development of modularized EVs with a special focus on the latest discoveries in the field of EV‐based nanodelivery systems.

## 2 Biogenesis and Release of EVs

### 2.1 Exosomes

One class of EVs is the Exos, “inward‐budding” vesicles that are generated in MVBs and secreted when the MVBs fuse with the cytomembrane.^55^, ^56^ The biogenesis and release of Exos is a complex symphony involving a series of factors, representatively including the endosomal sorting complexes required for transport (ESCRT), ALIX (also known as programed cell death 6‐interacting protein), phospholipase, vacuolar protein sorting‐associated 4 (VPS4), Rab GTPase proteins, sphingomyelinase, and ceramide.^57^, ^58^, ^59^

The classic mechanism involves a reverse‐topology membrane scission event mediated by an ESCRT‐dependent mechanism.^59^ The ESCRT machinery, which is formed by four ESCRT proteins (ESCRT‐0, ESCRT‐I, ESCRT‐II, and ESCRT‐III) and accessory proteins including ALIX and VPS4, assemble components of Exos together by forming intraluminal vesicles.^60^ ESCRT‐I is a claviform heterotetramer of tumor susceptibility gene 101, vacuolar protein sorting‐associated protein 28 (VPS28), the VPS37 Homolog (VPS37A/B/C/D), and one of the multivesicular body subunit 12A (MVB12A), MVB12B, or ubiquitin‐associated protein 1.^61^, ^62^ ESCRT‐II is a bifurcate heterotetramer consisting of one molecule each of VPS22 and VPS36 and two molecules of VPS25.^63^, ^64^, ^65^ The VPS28 subunit of ESCRT‐I and the VPS36 subunit of ESCRT‐II participate in the connection of the two complexes.^66^, ^67^, ^68^ ESCRT‐III directly participates in remodeling and abscission of the membranes; ultimately, VPS4, the recycling machine, extracts ESCRT‐III monomers from the assembly to recycle the ESCRT‐III subunits.^59^ An ESCRT‐independent mechanism involves the ceramide‐triggered budding of Exos into MVBs, which is preferentially dependent on certain cargoes.^69^ Another ESCRT‐independent mechanism involves tetraspanin (for example, CD63)‐mediated organization of particular proteins.^70^

**Figure 4:**
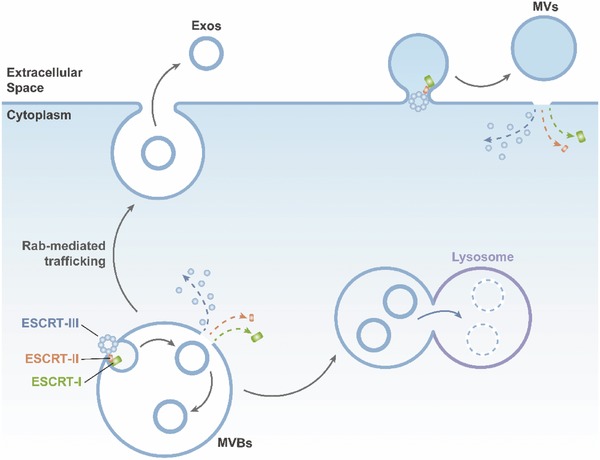
Biogenesis of Exos and MVs. After Exos are generated in MVBs by an ESCRT‐dependent mechanism, the MVBs fuse with the plasma membrane to release Exos or with lysosomes to recycle their contained lipids, proteins, and nucleotides.

After assembling, the MVBs fuse with the plasma membrane to release Exos, or with lysosomes to recycle their lipids, proteins, and nucleotides, to shut down an autocrine signal (**Figure** **4**).^71^ The process of fusing, and even the entire spatiotemporal traffic of Exos, is regulated by Rab GTPase proteins.^72^, ^73^

### 2.2 Microvesicles

Another class of EVs is the MVs, “outward‐budding” vesicles that are formed by budding from the surface of the plasma membrane and are simultaneously released.^55^ The mechanisms of MV biogenesis still require further study. One accepted mechanism mediated by ESCRT machinery (Figure 4), which is similar to the classic mechanism of Exos biogenesis, showed that MVs could be formed via the recruitment of ESCRT‐III proteins.^74^, ^75^, ^76^ In addition to the ESCRT‐dependent mechanism, it has been hypothesized that there is also an ESCRT‐independent mechanism of MV biogenesis. For instance, in cancer cells, the Rho‐associated kinase (ROCK)‐dependent signaling pathway appears to play an important role in generating MVs.^77^

## 3 Achievements and Limitations of Cell‐Free Therapy Using Native EVs

Scientists currently recognize that many biological functions, once considered to require the direct participation of stem cells, are actually mediated in a paracrine manner via the secretome, of which EVs are a vital component.^41^

One typical representative stem cell population, mesenchymal stem cells (MSCs), have been used in the treatment of diverse pathological conditions and are preferred because the procedure for their isolation and culture is easy, they can be isolated from a variety of tissues, including bone marrow, adipose, placenta, and umbilical cord blood, and their probability of neoplastic transformation or abnormal differentiation is relatively low.^78^, ^79^ In recent years, the therapeutic properties of MSCs have been considered to be due to the therapeutic effect of the secretome, especially EVs.^80^ A number of preclinical studies, including of ischemia, neurodegenerative diseases, fibrotic liver, and acute kidney injury, have explored the potential efficacy of MSC‐derived EVs in tissue regeneration.^81^, ^82^, ^83^ In addition, the immune‐suppressive capacity of MSC‐derived EVs, as a result of the proteins and RNAs they transport, has been recognized to regulate immunity via the release of anti‐inflammatory cytokines or modulation of the Toll‐like receptor signaling pathway.^84^

Other stem cells have shown a variety of common or unique biological functions. Human embryonic stem cell‐(hESC)‐derived EVs can induce dedifferentiation and pluripotency in their target cells.^85^ For instance, hESC‐derived EVs targeting retinal Müller cells can lead to alteration of the gene expression level and the epigenetic state by selectively transferring mRNA (Oct4 and Sox2), miRNA, and proteins, thus enhancing retinal regeneration.^85^ Moreover, endothelial progenitor cell‐derived EVs can activate the functions of endothelial cells to provide support for revascularization in injured tissues by transferring specific mRNAs and miRNAs.^86^ In addition, human cardiac progenitor cell‐derived EVs can promote the regeneration of injured heart tissue by increasing the proliferation of cells in/around damaged areas, including cardiomyocytes and endothelial cells, and inhibiting apoptosis of these cells.^87^ In addition, EVs of non‐nuclear origin, including platelets, also have incredible potential in regenerative medicine.^12^, ^88^

In tumor immunotherapy, the potency of dendritic cell (DC)‐EVs and C‐EVs, as components of a cell‐free tumor vaccine, has been receiving increasing attention.^89^, ^90^ Initially, DC‐EVs were found to play a role in antigen presentation by triggering a T cell‐dependent immune response, thus annihilating tumors.^89^ DC‐EVs were subsequently found to promote the activation of natural killer cells^91^ and B cells.^92^ Additionally, C‐EVs, after internalization by DCs, can further lead to the rejection of tumors via CD8+ T‐cell‐dependent antitumor effects.^90^

However, the latest opinions tend to consider that although EVs derived from different native source cells have their respective advantages, they cannot meet all the requirements of the increasing treatment needs of various diseases. For instance, synovial MSCs can enhance the proliferative and migratory abilities of chondrocytes but also seriously reduce the secretion of extracellular matrix.^12^ The solution to this problem is to enhance the expression level of miR‐140‐5p in EVs derived from synovial MSCs.^12^ It is easy to understand that EVs derived from native source cells will not serve as an “omnipotent catholicon” and thus artificial optimization of EVs, with the ultimate aim of modular design of EVs, will be the primary future directions of therapy.

## 4 Modular Design of the Lipid Bilayer

Lipophilicity is commonly utilized to modify EVs. Small lipophilic molecules, such as antioxidants, curcumin, and anticancer agents, can be passively loaded into EVs or EVMs by simple incubation.^52^, ^93^, ^94^ Drugs, including curcumin, show advanced stability and bioavailability when encapsulated compared to free‐state drugs and have been successfully used in preclinical research.^95^ Furthermore, siRNA, a frequently used genetic interference tool,^96^ can be conjugated with a lipid such as cholesterol to take advantage of its lipophilic properties to facilitate the packaging of siRNA into EVs.^97^

In addition to direct incubation with EVs or EVMs, small molecules can be effectively loaded into the parental cells to obtain EVs in conditioned medium. For instance, EVs or EVMs loaded with anticancer drugs were obtained after the parental cells were incubated with this drug.^52^, ^98^, ^99^ Furthermore, fluorescent lipophilic dyes are generally used as a tracer of EVs from conditioned medium after the parental cells have been labeled.^100^, ^101^

**Figure 5:**
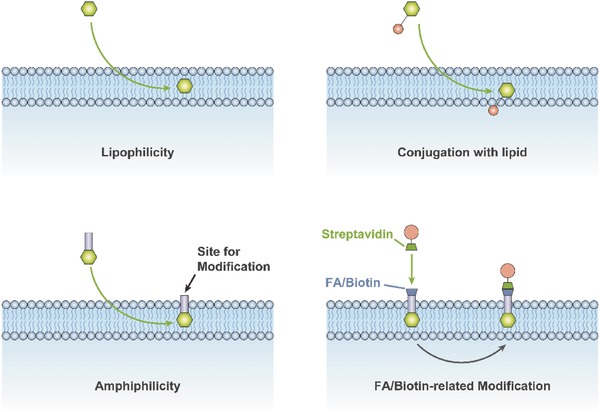
Methods based on lipophilicity and amphiphilicity for modular design of the lipid bilayer.

In addition to lipophilic drug loading, the lipid bilayer can also be profoundly modified, utilizing the amphiphilic molecules, to accomplish targeted delivery. Based on the fact that 1,2‐dioleoyl‐*sn*‐glycero‐3‐phosphoethanolamine–poly(ethylene glycol) (DSPE–PEG), an amphiphilic molecule, can self‐assemble into lipid bilayers, Zhang et al. obtained biotin‐ and folate‐ (FA)‐modified EVs (FA/biotin‐EVs) by culturing the source cells in medium supplemented with biotinylated DSPE–PEG (DSPE–PEG–Biotin) and FA‐modified DSPE–PEG (DSPE–PEG–FA). After conjugation with streptavidin‐conjugated iron oxide nanoparticles, the FA/biotin‐EVs were isolated from culture medium using a magnetic field and demonstrated magnetic steering targeting ability, while magnetic nanoparticles have great potential in diagnosis and treatment.^102^, ^103^, ^104^ We believe that such amphiphilic molecules will be at the frontier of targeted delivery of EVs, as well as studies of isolation and tracing. Conceptual diagrams showing the modular design of the lipid bilayer at present are shown in **Figure** **5**.

## 5 Modular Design of Surface Proteins

**Figure 6:**
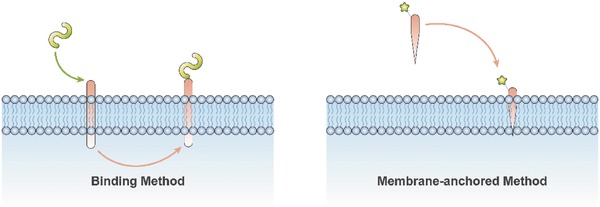
Two dominant strategies, including the “binding method” and the “membrane‐anchored method,” for the modular design of surface proteins.

One method commonly used to modify the surface proteins of EVs is called the “binding method,” which involves combining the “cargo” protein with a protein localized on the surface of the EVs by gene modification.^20^, ^105^, ^106^, ^107^ Already used binding segments include the C1C2 domain of milk fat globule‐EGF factor 8 protein,^105^, ^106^ the transmembrane domain of platelet‐derived growth factor receptor,^107^ and the extraexosomal N‐terminal of Lamp2b.^20^ Another method is called the “membrane‐anchored method” and involves anchoring the “cargo” protein to the membrane by fusing it to oligomeric membrane‐anchored proteins instead of certain EV‐related proteins.^108^ This method has been used in anchoring green fluorescent protein to EVs.^108^ Conceptual diagrams outlining these methods are shown in **Figure** **6**.

Research has shown that most nanosized particles are eliminated by the liver and kidneys, or phagocytosed by macrophages.^107^ The purpose of surface protein modification is to specifically change the in vivo distribution by regulating the targeting ability of EVs to not only enhance the specific effects on target cells but also to reduce off‐target effects. For instance, EVs expressing the rabies viral glycoprotein (RVG), a central nervous system‐specific peptide, via binding to the acetylcholine receptor have been shown to selectively deliver siRNA to neural cells, whereas EVs without RVG cannot.^20^ EVs with αv integrin‐specific arginine–glycine–aspartic acid peptides and loaded with doxorubicin selectively inhibited breast cancer progression, whereas unmodified EVs were mostly eliminated without affecting the tumor.^47^ Modified EVs have also demonstrated potential in specific targeting of oncogenic KRAS for tumor therapy.^109^ Recently, tumor necrosis factor (TNF)‐related apoptosis‐inducing ligand (TRAIL) was attached to the membrane of EVs, and these TRAIL‐armed EVs were found to inhibit tumor growth via transmission of proapoptotic signals.^110^

In tracing the target cells of EVs, transmembrane proteins fused to luciferase or fluorescent proteins in EVs can be used as tracers, and the luciferase or fluorescent activity of distant cells can be measured to trace the effects of EVs.^42^, ^111^

## 6 Modular Design of Internal Contents

As described above, the biogenic process of EVs occurs via protein‐mediated budding, and internalized substances, especially nucleic acids and proteins, are enveloped by the EVs for secretion. Because of these properties, functional improvement of EVs via modification of the internal nucleic acids and proteins could be realized through cell engineering, a field in which the currently available techniques are relatively advanced.

**Figure 7:**
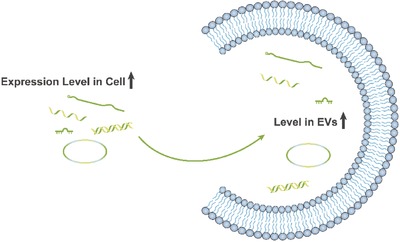
Modification of nucleic acids in EVs via cell engineering.

Recent studies have revealed that EVs can carry nucleic acids, including mRNA, miRNA, and other noncoding RNAs, such as long noncoding RNA (lncRNA).^1^, ^25^, ^112^ It has been proven that cells can package implanted mRNAs into EVs (**Figure** **7**), and transgenic protein expression is then induced after fusion with target cells.^113^ Furthermore, the protein expression induced by mRNA in EVs has been demonstrated to have valid bioactivity. A study showed that tumor growth was significantly reduced after transfection with EVs loaded with mRNA encoding a prodrug‐converting enzyme, along with systemic administration of the nontoxic prodrug.^114^

As for noncoding RNA, the miRNAs, which are comparatively well understood among all noncoding RNAs, have been proven to enhance the therapeutic effects of native EVs (Figure 7), provide new features, and reduce their side effects when specific miRNAs are loaded into EVs.^12^, ^43^, ^115^ Furthermore, in an unpublished study, we successfully packed lncRNA into EVMs and found that they could regulate the competing endogenous RNA (ceRNA) network of target cells.

**Figure 8:**
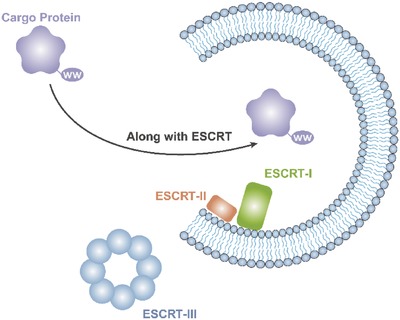
Transportation of intracellular proteins using ESCRT machinery.

To transport intracellular proteins, ESCRT machinery is commonly utilized. In a recent report, late‐domain (L‐domain) proteins, which are involved in the recruitment of ALIX, could be used to load specific exogenous intracellular proteins with a WW tag for recognition by L‐domain proteins into EVs (**Figure** **8**).^116^ Although ubiquitination and the ESCRT machinery have attracted the attention of many researchers and the underlying mechanisms have become increasingly clear, the packaging of proteins taking advantage of this feature still requires further investigation. We predict that utilizing the ESCRT machinery has the potential to be the major method of protein packaging.

In addition to the above functions, RNA‐modified EVs have also been used to trace the target cells of EVs. Lai et al. designed a practical tool, EVs containing fluorescently labeled mRNA to confirm the internalization of EVs by measuring the signal.^42^ Another effective and more proven method utilizes the Cre–LoxP system, in which transgenic mice express the LacZ reporter gene with a STOP sequence, which can be excised by Cre to subsequently exhibit LacZ expression after transfection with EVs containing Cre mRNA.^113^ This method has been successfully used several times.^117^, ^118^

**Figure 9:**
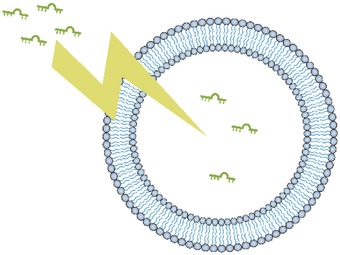
Direct transfer using physical or chemical methods, including electroporation.

**Figure 10:**
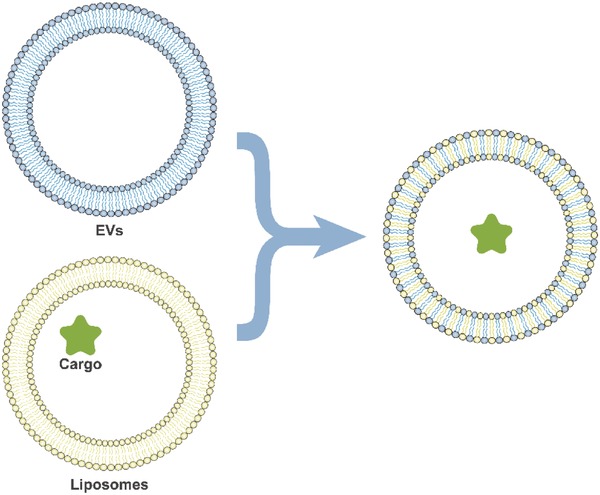
Integration of two vesicular systems, liposomes and EVs.

In addition to cell engineering, another serviceable strategy for creating modularized EVs is to directly transfer RNAs into EVs, including by electroporation. Leaders in this research area, such as Alvarez‐Erviti et al., have transferred siRNA into EVs via electroporation; subsequent experiments have demonstrated that these electroporated EVs could significantly inhibit expression of the target gene (**Figure** **9**).^20^ Electroporation to generate siRNA‐loaded EVs was successfully verified by follow‐up studies.^119^, ^120^ However, it appears that electroporation might not be efficacious for transferring all types of RNA directly into EVs without modifying the “source cells.”^107^ Simple electroporation might not be sufficiently efficient, and more in‐depth studies are needed.^121^ The latest theory suggests that integrating the advantages of two vesicular systems, liposomes and EVs, will be a potential solution to realize a superior siRNA delivery strategy (**Figure** **10**).^122^

Interestingly, there are several reports indicating the possibility that proteins can be directly transferred into EVs without modifying the “source cells.” A large protein, (*M*_W_ 240 kDa), has been effectively loaded into EVs (over 20% loading capacity) using several protocols, including sonication, extrusion, and permeabilization with saponin.^123^

Furthermore, the aforementioned methods could also be utilized for loading other imaging or therapeutic agents, such as gold nanoparticles.^123^

## 7 High‐Yield Tactics: Extracellular Vesicle‐Mimetic Nanovesicles

The primary technique to generate EVMs, crushing cells via serial extrusion through nanopore filter membranes with gradually reduced pore sizes (10, 5, and 1 µm) followed by isolation via two‐step density gradient ultracentrifugation, has been developed by Gho and co‐workers.^52^ In our unpublished work, we have added an extra extrusion step, using a 0.2 µm nanopore filter membrane immediately following the 1 µm nanopore filter membrane. The most fascinating feature of EVMs is that the production yield of EVMs is ≈120‐fold higher compared to EVs produced from the same number of cells.^52^

Although EVMs exhibit characteristics extremely similar to Exos,^53^, ^54^, ^124^ the actual identity of EVMs needs to be clarified by further investigation.

By analyzing the procedures used to generate EVMs, we conclude that it is highly likely that EVMs mainly begin as Exos in MVBs before they are naturally released or eliminated via lysosomes. This hypothesis could explain both the high yield and the similarity to Exos. The size of most normal mammalian cells is over 10 µm^125^, ^126^; therefore, extrusion through a 10 µm nanopore filter membrane would cause the cells to be destroyed and the organelles to be released. Mitochondria have a diameter of over 1 µm,^127^ so they could be subsequently removed with a 1 µm nanopore filter membrane, as could organelles such as the nucleus, endoplasmic reticulum, and Golgi apparatus, which are larger than mitochondria. The remaining Exos, proteins, and debris could then be distinguished by a two‐step density gradient ultracentrifugation due to their different densities. However, more in‐depth research is still needed. Once EVM production is proven to be a useful method to obtain a large quantity of EVs with rather simple steps, this method could be seamlessly connected to the modularized EVs to produce modularized EVs with high yields for clinical use or industrial production.

## 8 Outlook for Personalized and Precision Medicine

The foundation of personalized and precision medicine is the acquisition of a massive amount of personalized information from patients that can then be used to set up a precisely tailored therapeutic plan. For example, the application of human induced pluripotent stem cells (iPSCs), which could store personalized big data, could be an important component in personalized medicine—a patient's personalized database.^50^, ^51^ With the tremendous advances in “next‐generation” diagnostic techniques, thousands of mutations have been found and the origin of many diseases understood in greater depth.^128^ In addition, over the past few years, the arrival of liquid biopsy technology has made the acquisition of a database of tumors much easier, including DNA/chromatin mutations, methylation changes, and even information concerning C‐EVs.^129^, ^130^

Based on access to sufficient personalized big data, the key concept of precision medicine is the treatment or “ablation” of the pathological condition with limited damage to normal tissues.^49^ With adequate information from the database, it would be possible to assemble the needed cargo, including DNA, RNA, oligonucleotides, proteins, and drugs, into the modularized EVs using the technology described above, to maximize the therapeutic effect. Meanwhile, with highly customizable surface molecules, modularized EVs will have excellent targeting ability to avoid damage to normal tissues. As a consequence, modularized EVs will prove particularly effective in the field of prospective personalized and precision medicine.

## 9 Discussion and Conclusion

As two different kinds of lipid‐based delivery systems, liposomes and EVs show similar advantages while also having their own respective advantages and disadvantages. First, lipid nanoparticles exhibit high multifunctionality and biocompatibility among the nanoparticles that have been explored and have been proven to be safe and effective.^131^ EVs are good at carrying therapeutic agents, which could be genetically modified in parental cells by transfection.^132^ More precisely, EVs are serviceable for carrying proteins, especially membrane proteins for targeting delivery, and nucleic acids, which are hard to directly synthesize but easy to generate using bioengineered cells.^133^ However, liposomes are more appropriate for direct loading, as the adverse effects of directly loading contents into EVs including causing the aggregation of EVs^132^ or the possibility of relatively low efficiency, as mentioned earlier.^121^ A major reason for optimism is that the integration of liposomes and EVs will enhance their advantages and avoid their disadvantages.

In addition to EVs and liposomes, intelligent organic nanoparticles, such as semiconducting polymer nanoparticles (SPNs), are also available.^134^, ^135^, ^136^, ^137^, ^138^ SPNs constitute not only a new generation of organic nanoparticles for sensing and imaging^139^, ^140^, ^141^, ^142^, ^143^ but also a noninvasive remote control method with a near‐infrared absorbing property to regulate in vivo gene expression and cellular signals.^135^, ^142^, ^144^ What is particularly exciting is their potential in optimized cancer therapy.^145^, ^146^ The prospective integration of EVs and SPNs will have integrated superiority, such as targeting and controllability, just like the integration of liposomes and EVs, making them star performers in personalized and precision medicine.

Although the biogenesis of Exos and MVs are different, in most research into the function of EVs, their intracellular origin is not strictly distinguished. Instead, small‐sized EVs, isolated by 0.22 µm pore filters or ultracentrifugation, are regarded as Exos.^147^, ^148^ Therefore, other than explaining the biogenesis of EVs, we have avoided discussing them in detail in this review, similar to most EV‐related reviews.^1^ For therapeutic usage, Zhuang et al. have carried out pioneering work to provide a detailed comparison between the two main classes of EVs, Exos and MVs, as drug carriers, and they showed that Exos could be more effective due to their accumulation in the treatment area.^149^ Another kind of nanoparticle is the liposome, which has the longest circulation time, when with a particle size between 80 and 150 nm, instead of being quickly eliminated by the liver or spleen.^150^ If EVs behave in the same way, the superior effect of Exos (or small EVs) for therapeutic use could be due to the peak of Exos particle size distribution being in the 80–150 nm range.^28^, ^151^ However, further study of EV subtypes is still needed.

**Figure 11:**
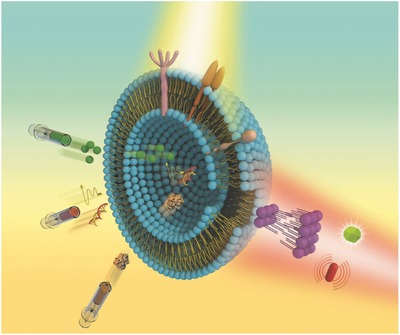
Concept and blueprint of the prospective modularized EVs.

In summary, the field of modularized EV‐based medicine holds significant promise for the realization of targeted and personalized drug delivery. As knowledge of the field further develops, scientists have begun to realize that EVs derived from native source cells cannot provide the answer for every condition and have certain deficiencies. Further study with respect to modification and optimization of EVs has gradually shown potential, just as a single spark is able to start a prairie fire. Almost all the components of EVs, including the lipid bilayer, surface proteins, internal proteins, and nucleic acids, can be modified and improved, and such modifications have been reported in increasing numbers. We expect that the ultimate research direction will be the “modular design” of every component of EVs, and the ability to obtain modularized EVs will be the ultimate therapeutic strategy (**Figure** **11**). If the essence of EVMs can be thoroughly understood, it will be easier to achieve industrial production of modularized EVs and thus meet the huge clinical demand for the needs of personalized and precision medicine in the future. However, there is still a long way to go to thoroughly study and more fully understand the biogenesis of EVs and to investigate more advanced, more efficient, and easier ways to achieve their “modular design”.

## Conflict of Interest

The authors declare no conflict of interest.
